# Multiscale and Multimodal Image Fusion. Coping with Differences in Scanned Area and Spatial Resolution for Raman/Fluorescence Images of Labeled Cells

**DOI:** 10.1021/acs.analchem.5c00492

**Published:** 2025-05-26

**Authors:** Albert Sicre-Conesa, Maria Marsal, Adrián Gómez-Sánchez, Pablo Loza-Álvarez, Anna de Juan

**Affiliations:** † Chemometrics Group, 16724 Universitat de Barcelona, Martí i Franquès, 1, Barcelona 08028, Spain; ‡ 172281 ICFO—Institut de Ciències Fotòniques, The Barcelona Institute of Science and Technology, Castelldefels, Barcelona 08860, Spain; § LASIRE (UMR 8516), Univ. Lille, CNRS, Laboratoire Avancé de Spectroscopie pour les Interactions, la Réactivité et l’Environnement, Lille F-59000, France

## Abstract

Multiscale and multimodal image fusion is a challenge derived from the diversity of chemical and spatial information provided by the current hyperspectral image platforms. Efficient image fusion approaches are essential to exploit the complementary chemical information across different zoom scales. Most current image fusion algorithms tend to work by equalizing the spatial characteristics of the platforms to be combined, i.e., downsampling pixel size and cropping noncommon scanned sample areas if required. In this work, a new image unmixing algorithm based on a flexible mathematical framework is proposed to enable working with all available image information while preserving the original spatial properties of every imaging measurement. The algorithm is tested on a challenging image fusion scenario of fluorescence and Raman images collected on labeled HeLa cells. The system is relevant from an analytical point of view, since smart fluorescence labeling allows profiting from the excellent morphological information without causing interferences in the rich chemical information furnished by Raman. From a data handling perspective, it offers a challenging multiscale problem, where the fast fluorescence imaging acquisition allows recording full cell images, and the slower Raman image acquisition is focused on scanning only relevant small regions of the cells analyzed. By applying the image fusion algorithm proposed, an improved morphological and chemical characterization of cell constituents in the full cell area is obtained despite the different spatial scales used in the original imaging measurements.

## Graphical abstract

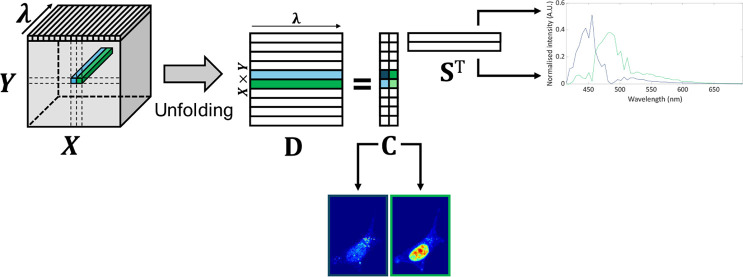

## Introduction

Hyperspectral imaging is a powerful analytical technology that combines chemical and spatial information, providing a wide range of insights across various scales and types of chemical (spectroscopic) features. Image fusion allows the connection of images from different modalities, facilitating the analysis of multiscale and multidimensional data. The combination of the information from multiple imaging techniques allows an integral morphological and chemical characterization of the sample components.^1,2^

Unmixing methods, such as Multivariate Curve Resolution-Alternating Least Squares (MCR-ALS), use the initial raw spectroscopic image information to provide a bilinear model formed by concentration maps and pure spectral signatures of the components within a sample, and can be adapted to handle image fusion scenarios.^3,4^ In the latter context, unmixing analysis works with a single data structure, called multiset, where the blocks of the different imaging measurements are interconnected. In classical image fusion scenarios, multisets need to share a common pixel mode among blocks, i.e., the imaging platforms fused should be associated with the same scanned area and need to present identical spatial resolution.^5^ These strong requirements need specific preprocessing that implies pixel downsampling in images with high spatial resolution and cropping big images to adjust to the platforms with the smallest area scanned.^6^ The global balance is that classical image fusion induces a loss of all nonequivalent information among image platforms. Besides, the downsampling operation has the negative effect of decreasing the spectral selectivity associated with methodologies of high spatial resolution, since the spectral information on neighboring pixels is combined, thus hindering the unmixing procedure.^5^

To overcome these limitations, incomplete multiset structures appeared and were formed by appending a core of fused information obeying the conditions of identical area scanned and pixel size among image platforms with additional blocks of noncommon specific information from the original images.^7,8^ These additional data blocks usually incorporate spectral information from higher spatial resolution images or from sample areas that were not scanned by all imaging modalities. Within an incomplete multiset, image-specific data blocks are connected with blocks of missing values because the rest of image platforms do not contain equivalent information.

Incomplete multiset analysis was initially tackled using a modification of the MCR-ALS algorithm that involved the combination of multiple partial models performed on complete submultisets of the initial data structure.^7,8^ This approach, while effective, had clear limitations when applied to real complex scenarios. Recently, a promising variant of the MCR-ALS algorithm has been introduced that provides a single bilinear model based on a flexible calculation procedure adapted to use only the available information in the incomplete multiset.^9^ The main values of this new approach are that a) this kind of algorithm adapts to the bilinear nature of the spectroscopic measurement and provides meaningful solutions because of the application of natural constraints, b) does not require imputation of missing observations and c) allows working with all available imaging information preserving the best possible spatial and spectral description of the measurements.

At this point, a qualitative comparison with other approaches may further justify the choice of this approximation over other options. Thus, a PCA modality that allows working with incomplete data sets without imputation has also been proposed by the authors for exploratory purposes but does not provide chemically meaningful and readily interpretable maps and spectral fingerprints.^10^ Likewise, deep learning methodologies might be an alternative, but the present approach is preferred because the spectroscopic signal of a hyperspectral image follows a linear behavior, and the use of more complex nonlinear methodologies is not required.

Up to now, the theoretical framework of the presented approach has been proven by showing a simple example as a proof of concept, but the algorithm has not been applied yet to any real complex system. An ideal scenario for testing this method involves the fusion of fluorescence and Raman images from labeled samples. The choice of this system is deliberate and opens an analytical way unexplored until now to combine the excellent morphological description of sample constituents provided by fluorescence labeling with the rich chemical description furnished by Raman spectroscopy.

From an analytical point of view, combining fluorescence and Raman images requires a smart labeling procedure that avoids fluorescence interferences in the Raman measurement. From a data handling perspective, the fusion of fluorescence and Raman images presents significant challenges. Raman images, as compared to fluorescence images, are typically chosen to be recorded with larger pixels (and therefore lower spatial resolution) to allow for an increased signal-to-noise ratio, with longer integration times. Furthermore, components of biological samples often exhibit similar Raman spectral signatures, which can make the unmixing task particularly difficult. To adapt to the natural acquisition speed characteristics of both imaging platforms, fluorescence images are acquired on the full cell area, while Raman images were focused on scanning small cell regions of particular interest. As mentioned above, such differences can be properly handled with the unmixing analysis algorithm adopted to handle incomplete data structures. The goal is to show that the analytical and data handling sides of this fusion approach improve both the morphological and chemical characterization of the samples analyzed, opening up new avenues in image analysis that have not been explored until now.

## Experimental Section

### Cell Culture and Sample Preparation

HeLa cells were cultured in Dulbecco’s Modified Eagle Medium (DMEM) (Thermo Fisher) with 10% Fetal Bovine Serum (FBS) (Capricorn Scientific), Glutamine 2 mM (Capricorn Scientific), and 1% penicillin–streptomycin (Biowest), at 37 °C and 5% CO_2_, until they were seeded. The cells were seeded on 25 mm round quartz coverslips, deposited in 6-well plates and cultured under the same conditions for 24 h. Then, they were fixed with 4% paraformaldehyde (PFA), and after 10 min, blocking and permeabilization of the cells was performed with Bovine Serum Albumin (BSA) and Triton X-100, respectively. BSA acts as a blocking agent that binds to nonspecific active sites of proteins present in the sample. By using BSA, specific binding of labeling agents only to targeted molecules is ensured. Triton X-100 is a surfactant used to lyse cells and permeabilise their membranes to facilitate the penetration of labeling agents. Lastly, labeling of the cellular compartments was carried out adding the following volumes of the concentrated fluorophore stock solution specified by each manufacturer to 1 mL of phosphate buffer solution (PBS) containing the cells: 1 μL DAPI (Thermo Fisher) to label the cell nucleus, 5 μL Lipi-Blue (DOJINDO) to label lipid droplets, and 15 μL Alexa FluorTM Plus 405 Phalloidin (Thermo Fisher) to label actin cytoskeleton. The labeled cells were incubated protected from light at room temperature for 1.5 h. The samples were washed with PBS after every preparation step. After the last incubation, the samples were stored protected from light at 4 °C until the measurements were made.

### Image Acquisition

To perform the measurements, the samples were placed in a magnetic chamber, covered with PBS, and vacuum-sealed with a glass coverslip. First, fluorescence images of individual cells were taken using a LEICA TCS SP8 STED 3X microscope (LEICA Microsystems, Mannheim, Germany). The light source used was a 405 nm laser with 20 μW of power focused through a 10× objective LEICA HC Pl Apo (NA = 0.4). This laser was suited to work with the labeling agents, which had their maximum excitation around 350 nm (DAPI), 375 nm (Lipi-Blue), and 405 nm (Alexa Fluor Plus 405 Phalloidin) (see Figures S1 and S2 for full spectral information in the Supporting Information). The detector used to record the emission spectra was a Gated HyD hybrid detector in photon counting mode. The emission spectra were gathered by point mapping with approximately 0.002 μs exposure time per pixel. The spectral emission range studied goes from 415 to 695 nm, with a spectral resolution of 5 nm and using 16 frame accumulations. The pixel size of the images was set to 0.2 × 0.2 μm^2^. The size of the images covering the whole cell area have an approximate size of 80 × 80 μm^2^. The images from the whole cells were identified as 1, 2, 3, 4, and 5 (Table 1).

**Table 1:** Overview of Fluorescence and Raman Microscopy Measurements for Each Sample

Cell	Fluorescence	Raman
1	1	-
2	2	-
3	3	-
4	4	4A, 4B
5	5	5A, 5B

Raman images were acquired using an inVia Raman Microscope spectrometer (RENISHAW, Gloucestershire, UK). The light source used was a 532 nm laser beam with 60 mW of power at the sample stage focused through a 60× water immersion objective Nikon (NA = 1.0) with an upright configuration. The laser wavelength was chosen to avoid excitation of the labeling agents and, hence, prevent interfering fluorescence contributions to the Raman signal, as shown in Figure S1. Spectra were collected by point mapping with 1 s exposure time per pixel. The spectral range recorded covered from 270 to 2015 cm^–1^, with a spectral resolution of 1.52–1.95 cm^–1^ depending on the Raman shift, with a deep depletion charge-coupled device (CCD) detector (RENISHAW RenCam), and an 1800 l/mm grating. The system was calibrated using an internal silicon sample with a known reference spectrum. The presence of a peak around 520.5 cm^–1^ was used to check the spectral channel accuracy. The pixel size of the images was 0.2 × 0.2 μm^2^ and they covered a small area of the cell that varied in size among the acquisitions. The images from the different areas scanned were identified as 4A, 4B, 5A, and 5B.

### Data Analysis

#### Image Preprocessing

For fluorescence images, a spatial binning was performed, i.e., the spectra of adjacent pixels were summed to create a larger pixel associated with a single spectrum in order to increase the signal-to-noise ratio of the recorded spectra, and to reduce the computational cost of the unmixing analysis. To reduce the size of fluorescence images keeping a sufficient spatial detail for proper unmixing, they were binned with a (2 × 2) factor. An example of a fluorescence image before and after binning is provided in Figure S3.

Spectral and spatial preprocessing were applied to Raman images. Cosmic peaks were replaced by interpolated Raman intensity values using those of the nearest spectral channels. After that, a filtering step based on the combination of Singular Value Decomposition (SVD) and Fourier transform was applied to reduce instrumental noise.^11^ The fluorescence baseline contribution was corrected by Asymmetric Least Squares (AsLs).^12^ Lastly, the region from 640 to 1750 cm^–1^ was selected for analysis. A plot of Raman spectra before and after preprocessing is included in Figure S4.

#### Multivariate Curve Resolution-Alternating Least Squares (MCR-ALS)

MCR-ALS is an iterative unmixing method that uses the information in the raw image spectra to provide the distribution maps and pure spectra of the image constituents,^3^ as shown in Figure 1.

**Figure 1:**
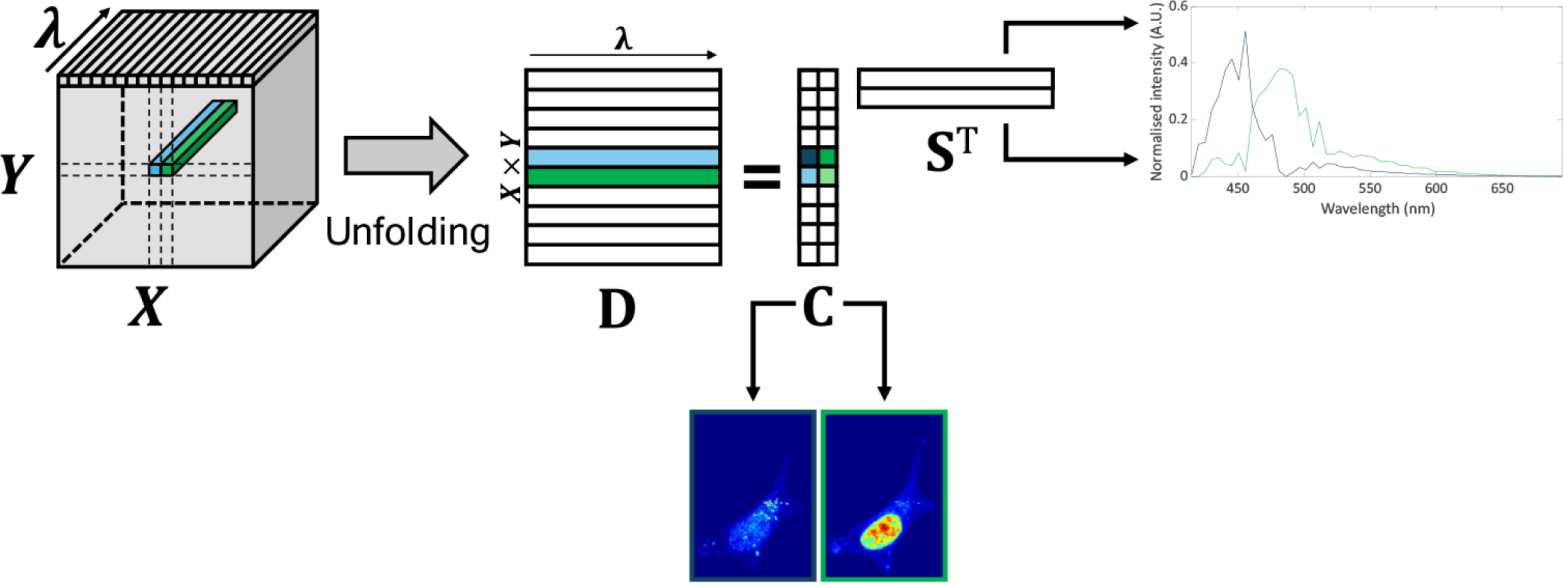
Data unfolding and final MCR-ALS solution from hyperspectral image analysis.

To do so, the initial 3D hyperspectral image (HSI), sized (pixels in X, pixels in Y, λ spectral channels) is unfolded into a data matrix **D**, sized (pixels in X × pixels in Y, λ spectral channels) which contains the spectra related to every pixel in the image. Then, MCR-ALS decomposes the matrix **D** into the bilinear model expressed in eq 1.

1 $$D=CS^{T}+E$$

Matrices **C** and **S** ^T^ contain the pure concentration profiles and spectral signatures of the different components of the sample, respectively, and **E** is the matrix containing the residuals of the MCR model. After obtaining an initial estimate of either **C** or **S** ^T^, the iterative optimization process starts by performing the alternating least-squares steps in eqs 2 and 3 for each iterative cycle until convergence is achieved.

2 $$C=\mathrm{DS}{\left(S^{T}S\right)}^{-1}$$

3 $$S^{T}={\left(C^{T}C\right)}^{-1}C^{T}D$$

The **C** and **S** ^T^ matrices are improved during the iterative process by including constraints suitable for a better unmixing. Some constraints available for HSI analysis are non-negativity, local rank constraints, and spatial constraints.^13^ These can be applied to **C** and **S** ^T^ separately or to specific sample components to enhance the quality of the MCR solutions and to reduce their ambiguity.

The convergence criterion for the iterative process can be defined by a maximum number of iterations or by a value related to the difference in the fit improvement between consecutive iterations, lack of fit (LOF). The parameters used to assess the quality of the fit between iterations (LOF and variance explained) are expressed in eqs 4 and 5

4 $$LOF_{1}\;\left(\%\right)=100\times \sqrt{\frac{\sum_{i,j}e_{i,j}^{2}}{\sum_{i,j}d_{i,j}^{2}}}$$

5 $$var\left(\%\right)=100\times \left(1-\frac{\sum_{i,j}e_{i,j}^{2}}{\sum_{i,j}d_{i,j}^{2}}\right)$$

where *d*_*i,j*_ is an element of the original data matrix **D** and *e*_*i,j*_ is the residual related to that element.

#### MCR-ALS for Image Fusion. Incomplete Multiset Analysis

An image fusion multiset consists of a set of concatenated images from the same sample recorded with two or more imaging platforms. In classical image fusion approaches, images must share the same area scanned and the same pixel size to be coupled and create a row-wise augmented multiset (Figure 2A).^3^ However, image platforms often show different spatial resolutions, and the scanned image area may not be identical among measurements. These differences induce a loss of information if classical image fusion is performed. Indeed, the need to have a common pixel mode in a row-wise augmented multiset requires adapting the pixel size of the images to the largest one present in the set, using a suitable binning factor.^5,6^ Some interesting areas of the images might also be lost in the process if some images frame larger areas of the sample than others as well.

**Figure 2:**
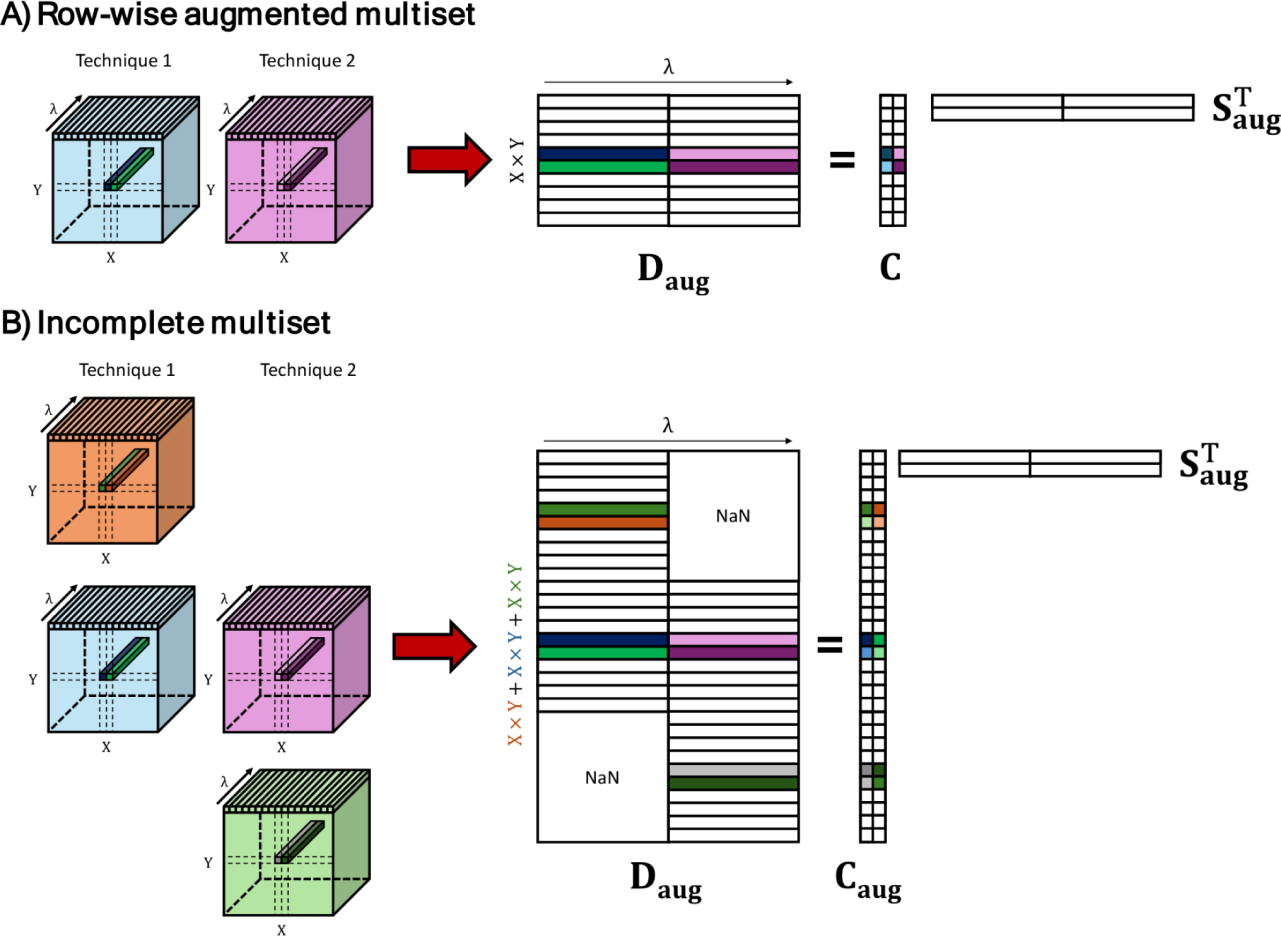
A) Example of a row-wise augmented multiset, in which the area scanned and pixel size is common for the images fused. On the right, an illustration of the unmixing of fused images. In this case, augmented spectral profiles are obtained. B) Example of an incomplete multiset, in which images that do not share the same areas, pixel size, and spectral information are appended. On the right, an illustration of the unmixing of an incomplete multiset, which provides augmented concentration and spectral profiles. NaN accounts for “Not a Number”.

To keep all the recorded information as in the original image measurements, an incomplete multiset can be built,^3^ such as the one shown in Figure 2B. This data set represents an image fusion scenario where two images acquired on the same sample by two techniques scanning different areas have to be analyzed. The common area scanned by the two images would be represented by the fused blocks in the central part of the multiset, whereas the image cube on top of Figure 2B would represent a sample area scanned only by Technique 1 and the bottom image cube an area scanned only by Technique 2. The top and bottom image cubes do not have an equivalent information in the other imaging technique and, hence, are connected with a block of missing information in the incomplete multiset. Apart from this illustrative example, many other kinds of incomplete multisets can be built when sample area and/or spatial resolution are not common to all images to be fused.^9^

Image multisets obey the basic bilinear model presented earlier in eq 1, which can be adapted depending on the situation, for instance, as in eq 6 for a row-wise augmented multiset (see Figure 2A), or as in eq 7 for a column and row-wise augmented multiset. The constraints mentioned earlier in this work can be applied when analyzing all kinds of multiset structures as well. In this case, constraints can be applied to the different submatrices forming **C** and **S** ^T^ in different ways, depending on the nature of the images or spectroscopic techniques that assemble the multiset.

6 $$\left[D_{1}D_{2}D_{3}...D_{K}\right]=C\left[S_{1}^{T}S_{2}^{T}S_{3}^{T}...S_{K}^{T}\right]+\left[E_{1}E_{2}E_{3}...E_{K}\right]=CS_{\mathrm{aug}}^{T}+E_{\mathrm{aug}}$$

7 $$\left(\begin{array}{c} D_{11}D_{12}D_{13}...D_{1L}\\ D_{21}D_{22}D_{23}...D_{2L}\\ D_{31}D_{32}D_{33}...D_{3L}\\ ...\\ D_{K1}D_{K2}D_{K3}...D_{\mathrm{KL}} \end{array}\right)=\left(\begin{array}{c} C_{1}\\ C_{2}\\ C_{3}\\ \vdots \\ C_{K} \end{array}\right)\left[S_{1}^{T}S_{2}^{T}S_{3}^{T}...S_{K}^{T}\right]+\left(\begin{array}{c} E_{11}E_{12}E_{13}...E_{1L}\\ E_{21}E_{22}E_{23}...E_{2L}\\ E_{31}E_{32}E_{33}...E_{3L}\\ ...\\ E_{K1}E_{K2}E_{K3}...E_{\mathrm{KL}} \end{array}\right)=C_{\mathrm{aug}}S_{\mathrm{aug}}^{T}+E_{\mathrm{aug}}$$

Incomplete multisets may also provide a complete bilinear model, even though there are missing blocks of information in the initial structure. The first approximation to solve the incomplete multiset problem was based on a combination of multiple factorizations on the submultisets that could be built from the initial incomplete multiset structure.^8^ Although this approach provided good results, it was complex to implement when many missing blocks exist in the initial data structure. Recently, a new proposal was made that allows a single factorization model by modifying slightly the least-squares calculations of **C** and **S** ^T^ during the optimization process.^9^ When missing values are present in a multiset structure, the **C** and **S** ^T^ matrices are calculated row by row and column by column, respectively, without considering the missing values in the calculations. The least-squares steps in this context can be described as adaptations of eqs 2 and 3 as follows:

8 $$c\left(i,:\right)=d\left(i,:\right)S{\left(S^{T}S\right)}^{-1}$$

9 $$s{\left(:,j\right)}^{T}={\left(C^{T}C\right)}^{-1}C^{T}d\left(:,j\right)$$

where eq 7 is used for the calculation of the rows of matrix **C**, and eq 8 is used to obtain the columns in **S** ^T^, respectively. Thus, the calculation of the *i*th row of **C**, named **c**(*i*,:), is done taking the nonmissing elements of the related row in **D**, **d**(*i*,:), and the part of the matrix **S** ^T^ related to the same nonmissing columns in **d**(*i*,:). Doing it row by row, the calculation of every row **c**(*i*,:) is adapted to the available information in the related row in **D**, **d**(*i*,:), which can be changing across the incomplete multiset. A similar reasoning can be done for the calculation of every *j*th column of **S** ^T^, **s**(:,*j*)^T^, which will be done using the nonmissing elements in the related column of **D**, **d**(:,*j*) and the part of the matrix **C** related to the same nonmissing rows in **d**(:,*j*) (see Figure S5 for a graphical explanation of these calculations). As a result, full **C** and **S** ^T^ matrices are obtained, onto which constraints can be applied as in the analysis of complete multiset structures.^9^

The MCR-ALS algorithm was used to analyze complete data sets via the graphical user interface MCR-ALS GUI 2.0, which runs under MATLAB environment.^14^ To perform preprocessing operations and the analysis of incomplete multisets, in-house developed MCR-ALS MATLAB routines suited to this kind of data structures were used.^9^ Routines are available on request.

## Results and Discussion

Prior to the incomplete multiset analysis of fused Raman and fluorescence images, separate column-wise augmented multisets assembled either appending only fluorescence or only Raman images of the cells studied were analyzed by MCR-ALS.

For the multiset formed by fluorescence images, the number of components to be selected was previously known owing to the number of labeling agents used. However, in the Raman analysis, the selected number of components was based on an initial Singular Value Decomposition analysis followed by testing few models with different numbers of components.^15^ The chemical meaningfulness of the recovered distribution maps and spectral signatures and the lack of fit obtained for every model were the criteria considered to select the definitive model.

In all cases, a SIMPLISMA-based algorithm was used to obtain the initial spectral estimates.^16^ Non-negativity was applied to the concentration and spectral profiles of both multisets since fluorescence and Raman spectra cannot have negative values.^17^ In the Raman analysis, the correspondence of species constraint, linked to the definition of presence or absence of some constituents in the images analyzed, was used since the scanned area of some of the images did not include certain cellular components.^18^

Table 2 summarizes the main MCR-ALS results obtained in all multisets analyzed. An example of the distribution maps and spectral signatures retrieved from the fluorescence and Raman analyses are shown in Figure 3A,B, respectively. The complete results for these analyses are displayed in Figures S6 and S7.

**Table 2:** Summary of the Results Obtained from the Image Multiset MCR-ALS Analyses

Multiset	Techniques	No. of components	LOF (%)	Explained variance (%)
1	Fluorescence	3	16	97
2	Raman	5	3	100
3	Fluorescence, Raman	6	10	99

**Figure 3:**
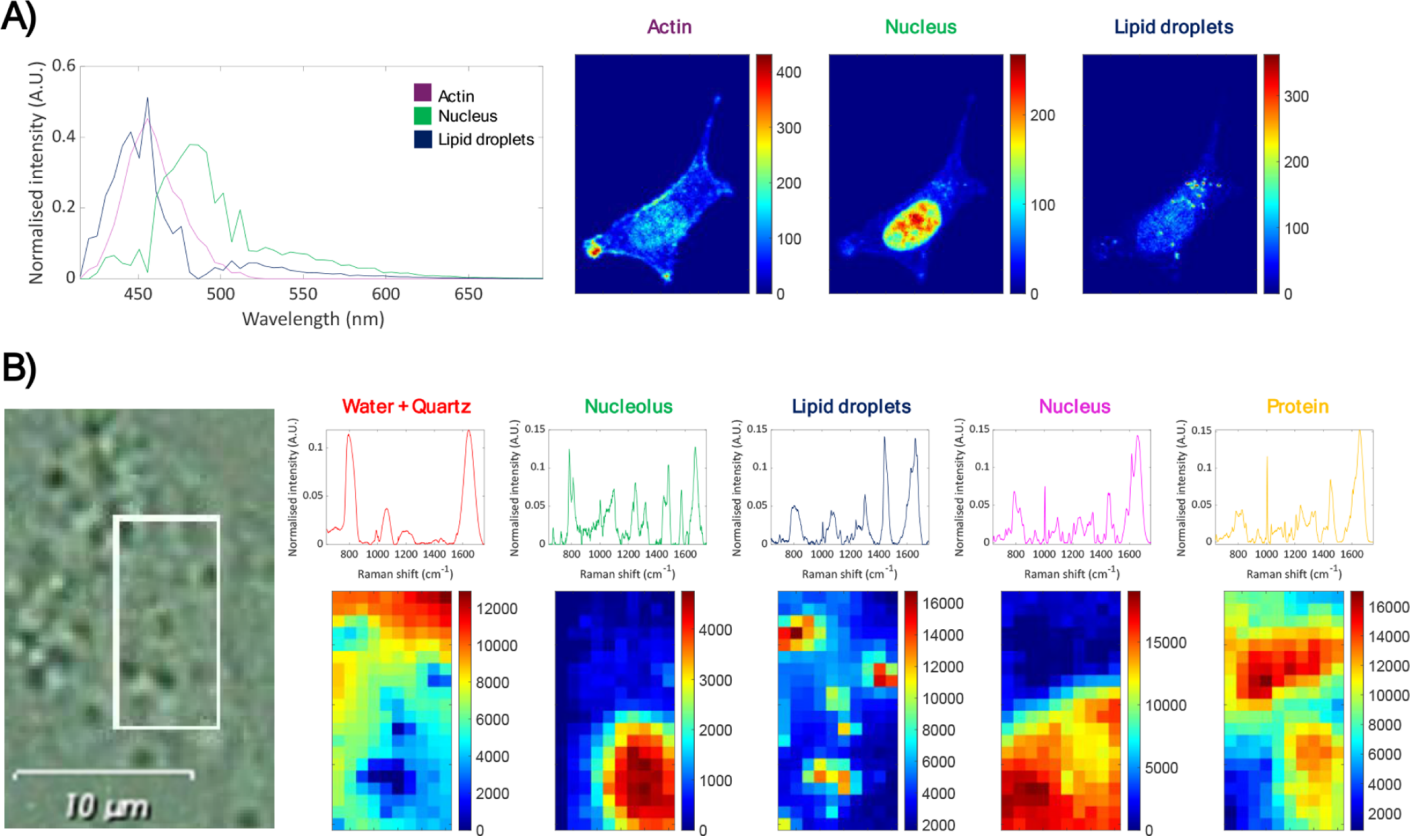
A) Distribution maps and spectral signatures retrieved from fluorescence multiset MCR analysis. B) Distribution maps and spectral signatures retrieved from Raman multiset MCR analysis. On the left, gray optical image related to the scanned region.

Three components were detected in fluorescence images, matching the number of structures labeled, i.e., the nucleus, the actin cytoskeleton, and lipid droplets. In Raman images, five components were detected and assigned: nucleus, nucleolus, lipid droplets, protein enriched regions, and sample support (water and quartz). For further information on the Raman features used to assign the components see Table S1.

## Incomplete Multiset Analysis

Figure 4 shows the structure of the incomplete multiset used for fluorescence and Raman image fusion. The first step in the construction of the incomplete multiset was the fusion of fluorescence and Raman images from the small cell areas identified as 4A, 4B, 5A and 5B, scanned by the two kinds of platforms. The small Raman areas scanned were selected by visual inspection of the optical image provided by the instrument and included regions containing the essential biological constituents of the cell, i.e., nucleus, cytoplasm and lipid droplets, that were showing distinct spatial patterns easily identifiable by both techniques to facilitate the step of image alignment among platforms. To perform the image fusion procedure, the large fluorescence images were cropped to approximately match the measured Raman areas. Afterward, to improve the coregistration (alignment) procedure of both kinds of images, the images recorded by the two platforms were downsampled by binning (from 0.2 × 0.2 μm^2^ to 0.8 × 0.8 μm^2^ pixel size) to increase the signal-to-noise ratio of the measurement. To start the image alignment, there are many possibilities regarding the initial information used. Among those, an excellent option is selecting distribution maps of image constituents with a distinct spatial pattern obtained by the image unmixing procedure performed separately on every technique^6^. In this study, the distribution maps related to the lipid droplets obtained in the previous fluorescence and Raman MCR multiset analyses were used as the reference for image alignment. This choice was justified because the pattern of the vesicles was clearly displayed in both fluorescence and Raman maps. Once the shift and rotation aligning parameters were obtained and applied to perform the image matching,^6^ the pixel spectra of the binned fluorescence and Raman images were concatenated, and the central part of the incomplete multiset (framed in black in Figure 4) was built. To enlarge the incomplete multiset, the spectra from the fluorescence images of complete cells 1, 2, 3, and 4 (framed in blue in Figure 4) were included on top of the multiset and the spectra of the Raman 4A, 4B, 5A, and 5B with the initial spatial resolution were appended at the bottom of the multiset. The fluorescence and Raman images appended to the central Raman/fluorescence fused core preserved their original pixel mode and, hence, did not have equivalent information on the complementary technique and were concatenated to blocks of missing information. This way, the incomplete multiset was constructed using all the available recorded image information.

**Figure 4:**
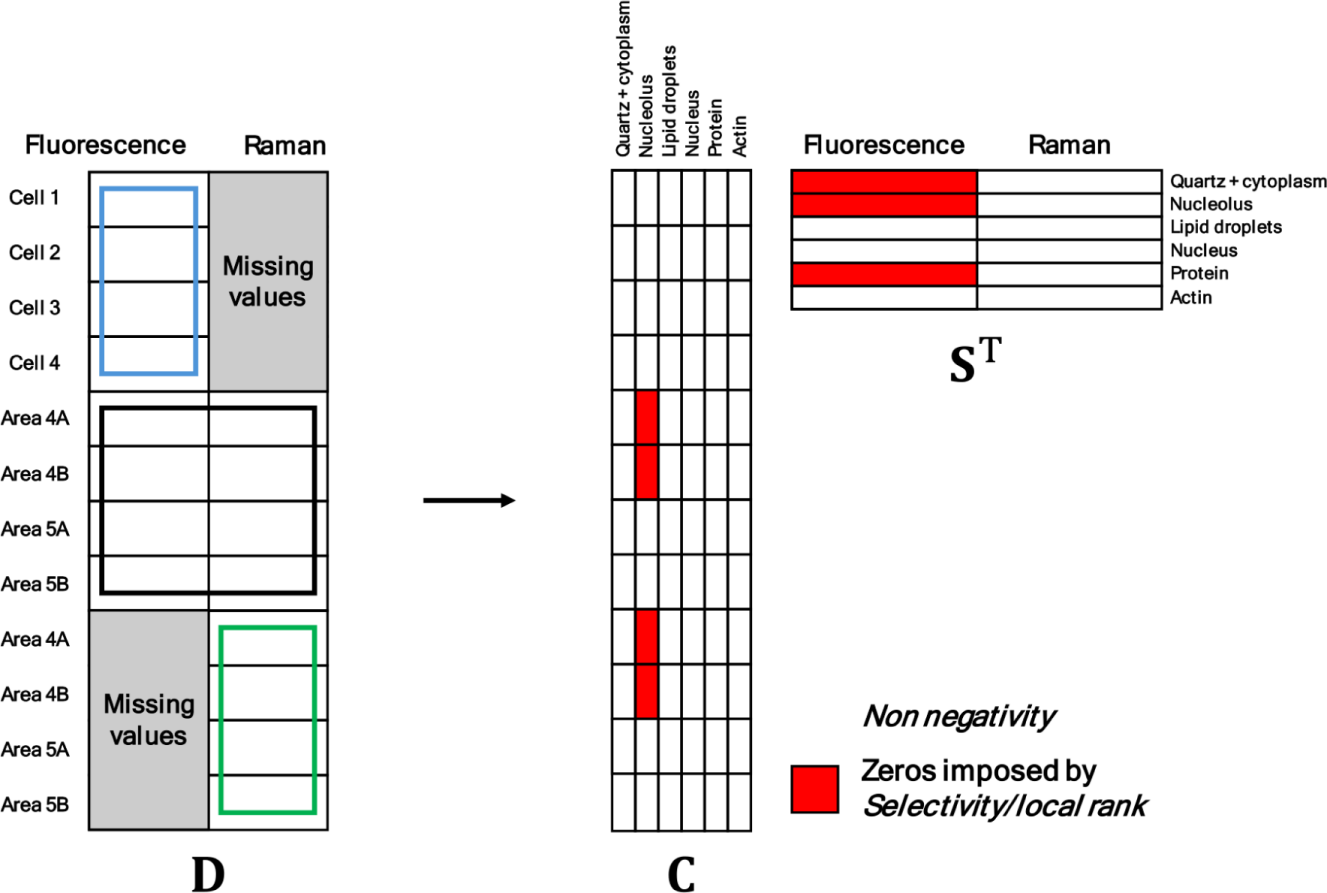
Scheme of the incomplete data structure and the constraints applied after unfolding the data matrix into **C** and **S** ^T^. The red parts are concentration profiles or spectral signatures set to zero because of the absence of certain components in samples (red blocks in **C**) or null signal in some techniques for some components (red blocks in matrix **S** ^**T**^).

The incomplete multiset was analyzed by MCR-ALS, providing a matrix of augmented concentration profiles, related to the different image blocks connected, and extended fluorescence-Raman spectral signatures for the different cell components. As initial spectral estimates, combined pure spectral profiles from previous fluorescence and Raman MCR-ALS analyses were used. The constraints used were non-negativity for the concentration and spectral profiles and equality constraints to indicate the absence of some cell components in certain samples or the null signal in certain imaging techniques for specific cell components. Equality constraints act setting to zero the absent components in samples or the null signal for some techniques across all MCR iterations. More specifically, the correspondence of species (equality constraint in matrix **C**) was used to indicate the absence of the nucleolus component in 4A and 4B images (see red blocks in the **C** matrix in Figure 4), and equality constraints in matrix **S** ^T^ were used to indicate the null fluorescence signal for nonfluorescent components, i.e., the sample support, the nucleolus,^19^ and the protein-enriched regions (see red labeled spectral channels in the **S** ^T^ matrix in Figure 4). Applying equality constraints that are consistent with the nature of the system, the spatial and spectral results reflect the actual behavior of the samples and the rotational ambiguity of the final MCR solution is clearly decreased. The values of lack of fit and explained variance shown in Table 2 are satisfactory, which indicates that constraints used in the MCR-ALS model are obeyed by the system analyzed.

An example of the pure distribution maps of the central core of fused images (i.e., maps related to the black framed area in Figure 4), and the spectral signatures obtained in the MCR-ALS analysis of the incomplete multiset are shown in Figure 5.

**Figure 5:**
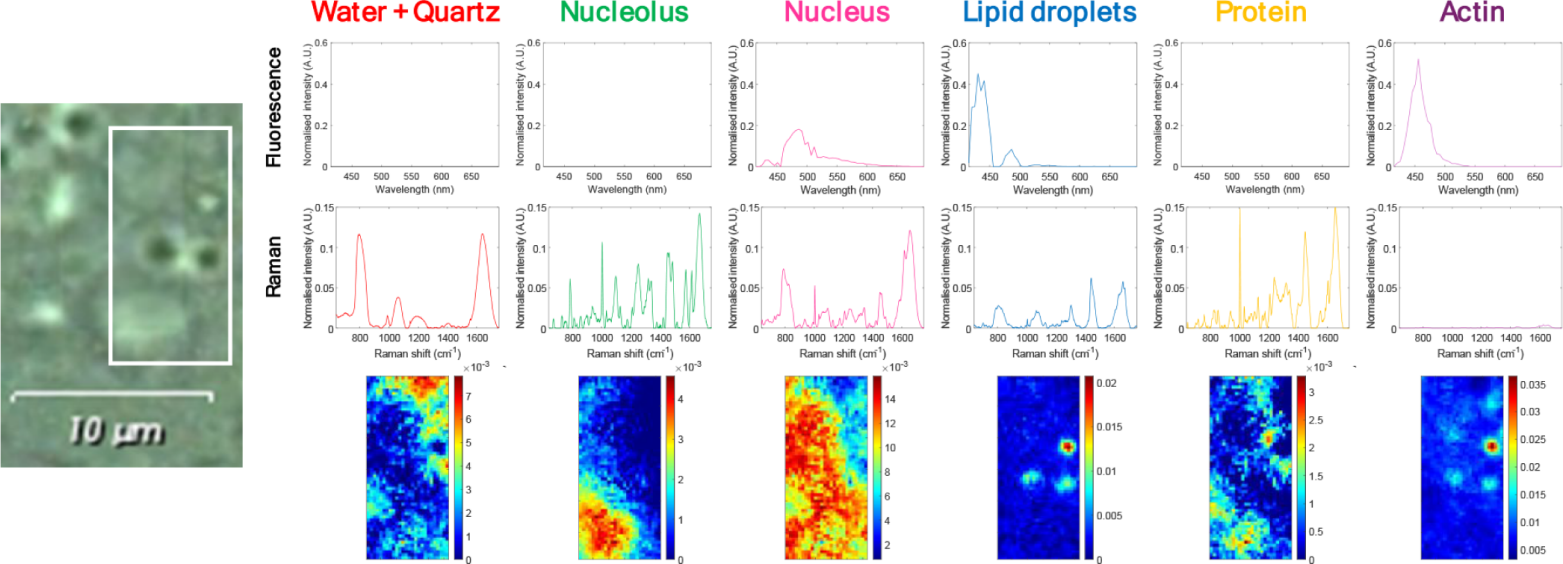
MCR-ALS results of the incomplete multiset analysis for the different cellular components. Top two rows: fluorescence and Raman pure spectral profiles. Bottom row: related pure distribution maps.

For the full results of the fused images see Figure S8. The distribution maps obtained for the fluorescence images (related to the framed blue area in Figure 4) are displayed in Figure S9.

The component assigned as nucleus was first identified thanks to its distribution map and the combined fluorescence and Raman pure signature. The nucleus is the biggest cellular organelle, and it is involved in metabolic functions such as DNA synthesis and transcription, among many others. Therefore, it is rich in protein and nucleic acids, which agrees with the Raman signature obtained.^20^

The actin cytoskeleton was only elucidated in fluorescence images using AlexaFluor 405 to label actin filaments. The area shown in the distribution maps spatially matches the literature. This component is found as stress fibers defining the cell shape, and on the outside, as thin protrusions called filipodia.^21^ The Raman/fluorescence fusion allows the detection of this cellular component, which could not be appreciated only with Raman, as confirmed by the flat null signal related to this technique in Figure 5.

The component found inside the nuclear region only in Raman images was assigned to the nucleolus. Its main function is the biogenesis of rRNA and ribosomal subunits; therefore, as can be seen with the related Raman features, is a region rich in RNA, DNA, and protein.^22^

Lipid droplets were first seen in fluorescence images using Lipi-Blue to label them. Then, a component was retrieved in Raman analysis. Moreover, the chemical lipidic nature of this component was confirmed by the Raman signature with features associated with neutral lipids, wax esters, and sterol esters.^23^

Raman analysis provided a component mainly located inside the nucleus and surrounding the nuclear area. The related spectra showed features that indicated protein and RNA presence. This protein-enriched regions could be related to structures where protein synthesis is carried out, e.g., ribosomes belonging to the rough endoplasmic reticulum, membrane-bound to the nucleus.^24^

Lastly, the component assigned as the sample support only had Raman signal and the distribution maps tell that it is mainly present in regions with water and outside the cell.

As seen in the description above, distribution maps and spectral profiles were retrieved for all the components elucidated by one or the two imaging techniques. Thus, this kind of analysis allows characterizing all the components at the same time regardless of whether they are identified only with a certain platform.

There are some clear general benefits associated with this integral image fusion approach, namely:

a) Every component is related to concatenated fluorescence and Raman signatures, facilitating chemical identification. These extended spectral signatures provide information on the correlated features of the two techniques due to the natural structure of matrix **S** ^T^ in the bilinear model.
b) The extended spectral signatures are valid to chemically characterize the components present in the fused scanned areas and, by extension, the same components in sample areas scanned by a single platform. In this case, the Raman signatures related to labeled components are valid not only for the small Raman scanned areas, but also for the whole cell images, i.e., the maps of the entire cells 1, 2, 3, and 4 shown in Figure S9 and only scanned by fluorescence are also associated with the Raman fingerprints of the components resolved. This is a great advantage because cellular structures present in different locations can be chemically characterized selecting interesting regions to be measured with Raman while being fully spatially defined by fluorescence images recorded on the complete cell.
c) The morphology of the distribution maps is improved when defined by two techniques. In this example, the image fusion improved the definition of the shape of the distribution maps due to the excellent morphology definition associated with fluorescence-labeled components.

The satisfactory results evidence that the implementation of this MCR-ALS strategy for incomplete multiset analysis of complex biological samples is viable. The application of this MCR modification is a suitable alternative to circumvent the problems related to multiscale and multiplatform image fusion.

## Conclusions

Multiscale image fusion is a challenging task arising from the need to integrate measurements obtained from imaging platforms that differ in the nature of their spectroscopic signals. These differences result in a large diversity of spatial resolutions and image acquisition speeds and, together with the growing use of multimodal microscopes, clearly necessitate establishing protocols for effectively handling these complex image fusion procedures.

This work demonstrates that progress in multiscale image fusion goes together with tailored analytical and data-handling solutions. From an analytical perspective, smart fluorescence labeling has enabled combining the excellent morphological characterization of labeled sample components in fluorescence images with the rich chemical information provided by Raman imaging.

From a data-handling point of view, improving unmixing methodologies to address differences in scanned areas and spatial resolution between imaging platforms is critical. Incomplete multisets offer a promising approach to integrate all available image information in its original form into a single data structure, preserving both platform-specific and common knowledge among platforms. To interpret these complex incomplete multisets, the newly developed MCR-ALS framework that works using a single bilinear factorization model appears as the most flexible and computationally simple option to accommodate all possible patterns of spatial variation across imaging platforms. The *modus operandi* of the algorithm presented in this work is extendable to scenarios of image fusion involving different imaging techniques and samples of different nature, as long as the underlying bilinear model of the measurements is fulfilled, which is generally the case after the suitable spectral preprocessing. The application extends easily to other kinds of samples related to characterization of new materials, cultural heritage or industrial contexts. This generalization of the approach is understandable since MCR-ALS was already used successfully in all these image fusion contexts when no missing observations were present. The only limitation found in this methodology is linked to the natural characteristic of any soft-modeling methodology, i.e., in image blocks specifically linked to a single technique, no retrieval of maps of components with null signal is possible.

In the example presented in this work, the application of this unmixing methodology significantly improves the characterization of sample components. The resulting maps exhibit a better morphological definition, while the extended multitechnique spectral signatures improve component identification, delivering a more comprehensive understanding of the samples studied.

## Supplementary Material



## Notes

Fluorescence spectral information of the labelling agents, a graphical explanation of the modified least squares steps in incomplete multiset MCR-ALS analysis, full results of fluorescence and Raman individual MCR-ALS analyses, and fluorescence distribution maps (PDF)
