# Supplementary material for: Multiscale and Multimodal Image Fusion. Coping with Differences in Scanned Area and Spatial Resolution for Raman/Fluorescence Images of Labeled Cells
Source: Anal Chem. 2025 May 26;97(22):11554–62. doi: 10.1021/acs.analchem.5c00492 (PMC12163868; doi:10.1021/acs.analchem.5c00492)
Supplement: Supplementary file 1 [file ac5c00492_si_001.pdf]

Supporting information

Multiscale and multimodal image fusion. Coping with differences in scanned area and spatial resolution for Raman/fluorescence images of labelled cells

Albert Sicre Conesa[1], Maria Marsal[2], Adrián Gómez-Sánchez[1,3], Pablo Loza-Álvarez[2], Anna de Juan[1,*]

[1]Chemometrics Group, Universitat de Barcelona, Martí i Franquès, 1, 08028 Barcelona.

[2] ICFO—Institut de Ciencies Fotoniques, The Barcelona Institute of Science and Technology, 08860, Castelldefels (Barcelona), Spain

[3]LASIRE (UMR 8516), Univ. Lille, CNRS, Laboratoire Avancé de Spectroscopie pourles Interactions, la Réactivité et l'Environnement, Lille, France

(*) Corresponding author: anna.dejuan@ub.edu

Abstract

The supporting information includes fluorescence spectral information of the labelling agents used in figures S1 and S2. Figures S3 and S4 show images and/or spectra before and after preprocessing in fluorescence and Raman images, respectively. A graphical explanation of the modified least squares steps in incomplete multiset MCR-ALS analysis is displayed in figure S5. Full results of fluorescence and Raman individual MCR-ALS analyses can be consulted in figures S6 and S7, respectively. Table S1 contains the Raman features used for component identification in Raman and image fusion analyses. Figure S8 displays all the results related to the fused images from the incomplete multiset analysis. Figure S9 shows the fluorescence distribution maps recovered from the incomplete multiset analysis.

Labelling agents excitation/emission profiles

Excitation profiles

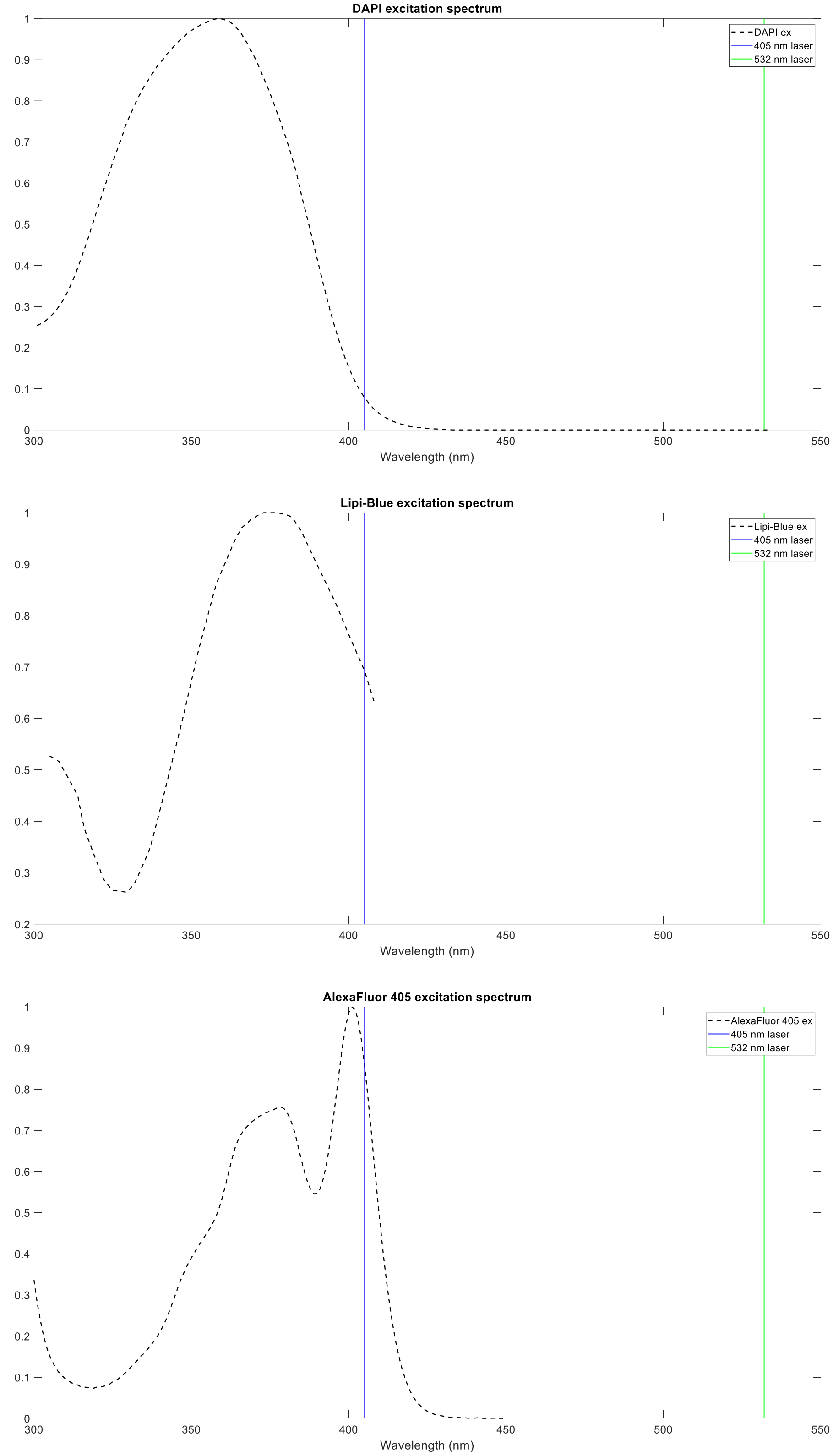

Figure S1. From top to bottom: excitation profiles of DAPI, Lipi-Blue, and AlexaFluor 405. The blue and green lines account for the 405 nm laser used in fluorescence, and the 532 nm laser used in Raman measurements, respectively.[25]

Emission profiles

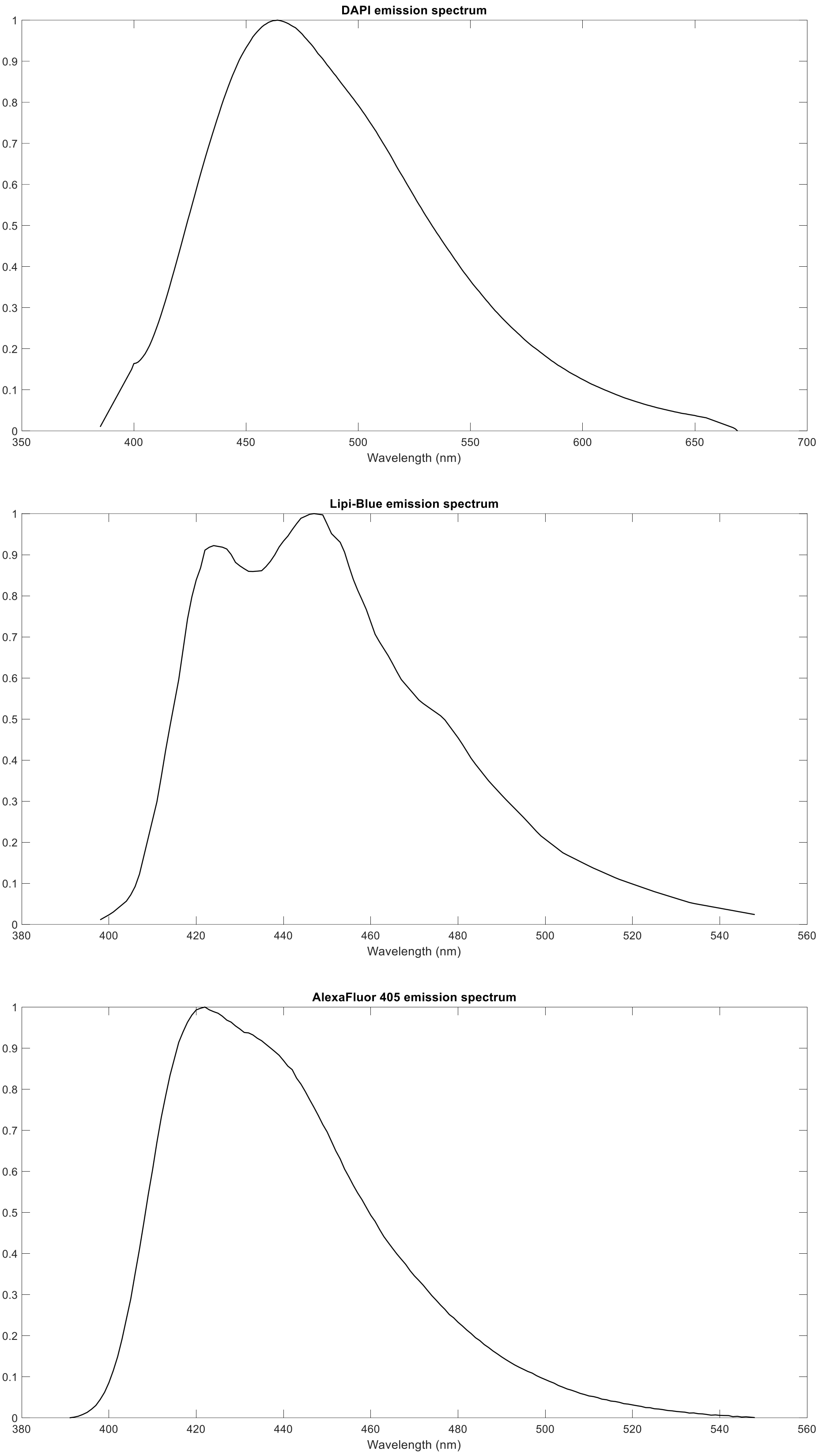

Figure S2. From top to bottom: emission profiles of DAPI, Lipi-Blue, and AlexaFluor 405.[25]

Image preprocessing

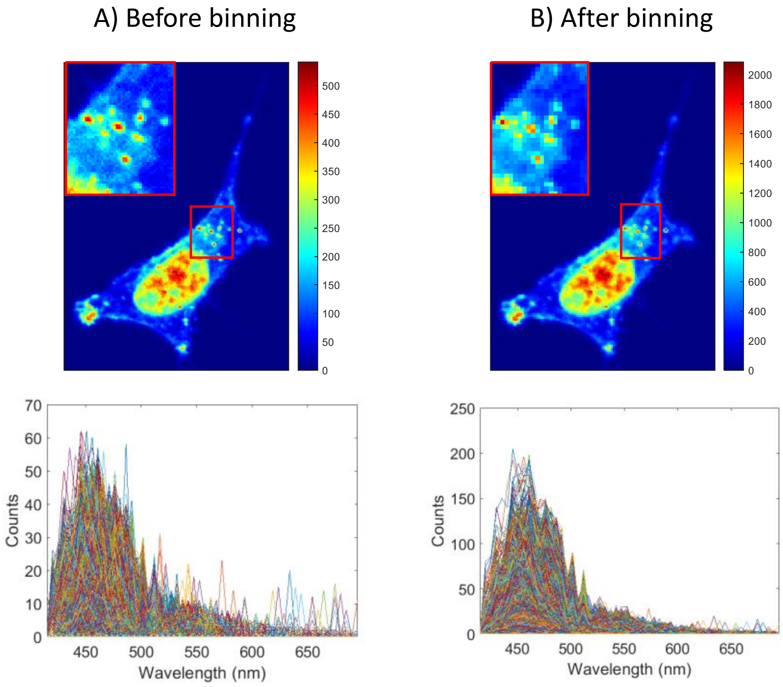

Figure S3. a) Global intensity map (the sum of the intensities of all spectral channels of each pixel spectrum) (top plot) and pixel fluorescence spectra (bottom) of the raw image of a HeLa cell. b) Analogous information to a) after binning.

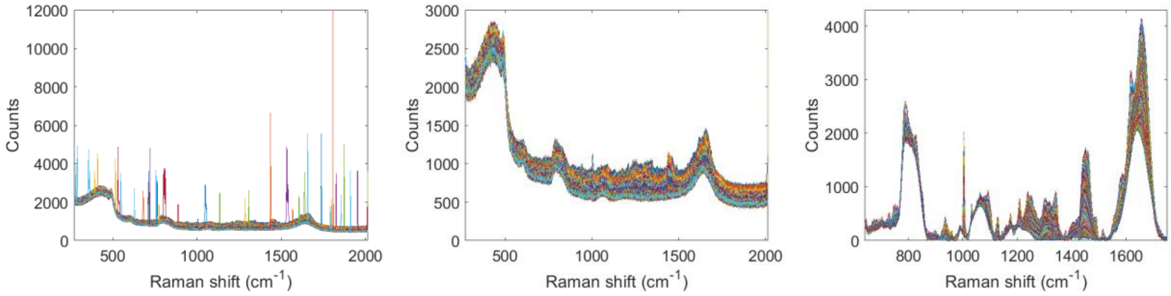

Figure S4. On the left, raw Raman spectra. On the middle, Raman spectra without cosmic peaks. On the right, fully preprocessed Raman spectra after baseline correction.

Least squares calculations in incomplete multiset analysis

Figure S5 shows how the least squares operations are carried out in the single factorization model approach. In Figure S5 a) the row-by-row calculation of $\mathbf{C}$, formed by submatrices $\mathbf{C}_1$ and $\mathbf{C}_2$, is displayed. $\mathbf{C}_1$ is related to rows in $\mathbf{D}$ matrix that do not contain any missing information and, therefore, the total $\mathbf{S}^{\mathrm{T}}$ matrix could be used in the least-squares calculation step (left plot in section a)). However, $\mathbf{C}_2$ is associated with rows in the $\mathbf{D}$ matrix that do not contain the full spectral information ($\mathbf{D}_3$). Hence, only the $\mathbf{S}_1^{\mathrm{T}}$ block on the spectral profiles could be used in the $\mathbf{C}_2$ least-squares calculation (right plot in section a)). Similarly, in Figure S5 b) the column-by-column calculation of $\mathbf{S}^{\mathrm{T}}$, formed by $\mathbf{S}_1^{\mathrm{T}}$ and $\mathbf{S}_2^{\mathrm{T}}$ blocks, is displayed. The $\mathbf{S}_1^{\mathrm{T}}$ block is related to columns in matrix $\mathbf{D}$ that do not lack any information and, hence, the complete $\mathbf{C}$ matrix could be used in the least-squares calculation (left plot in section b)). By contrast, $\mathbf{S}_2^{\mathrm{T}}$ is associated with columns in the $\mathbf{D}$ matrix that contain missing values. Now, only the $\mathbf{C}_1$ matrix could be used in the $\mathbf{S}_2^{\mathrm{T}}$ least-squares calculation (right plot in section b)).

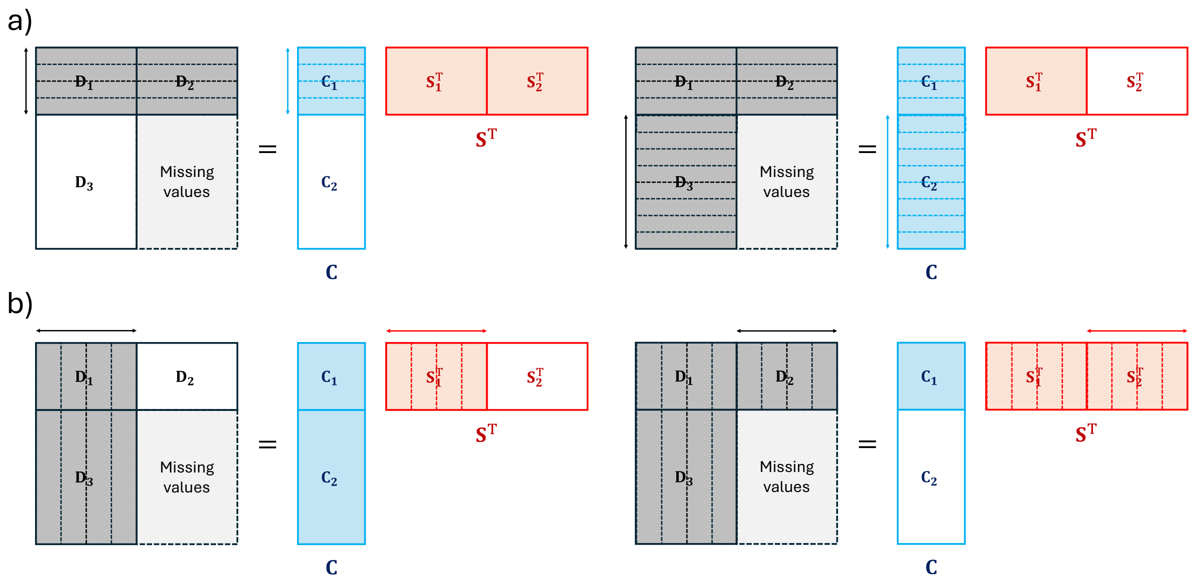

Figure S5. MCR analysis of an incomplete multiset with a single factorization model. a) $\mathbf{C}$ and b) $\mathbf{S}^{\mathrm{T}}$ calculation. Reprinted from ref.[9]

Fluorescence multiset results

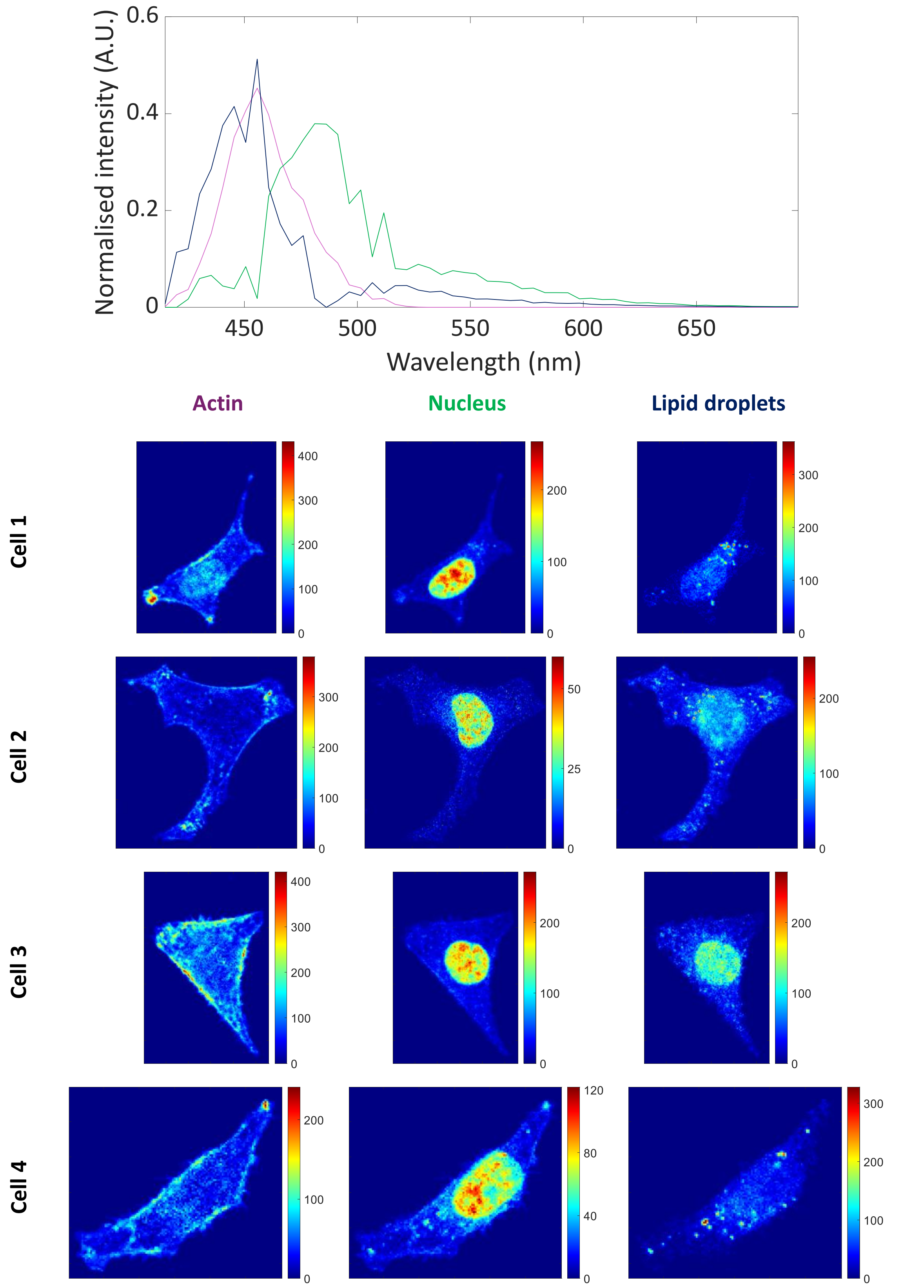

Figure S6. Results retrieved from the fluorescence multiset MCR-ALS analysis. Top, spectral signatures of the three labelling agents. Bottom, distribution maps of actin, nucleus, and lipid droplets.

Raman multiset results

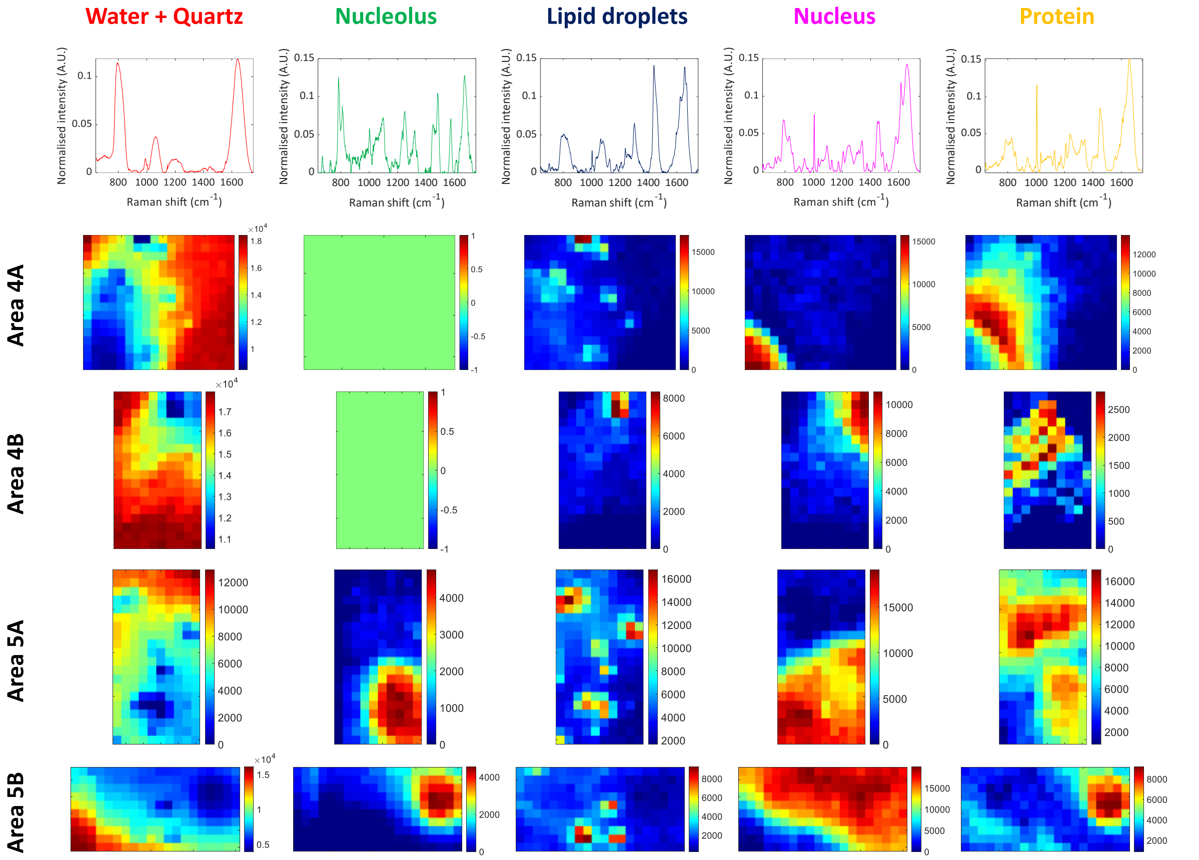

Figure S7. Results retrieved from the Raman multiset MCR-ALS analysis. Top, spectral signatures of quartz and water (sample support), nucleolus, lipid droplets, nucleus, and protein enriched regions. Bottom, distribution maps of the components elucidated. Green maps represent absent components in Areas 4A and 4B.

Raman features used in component assignation

Table S1. Raman features used in component assignation.

Component	Peak (cm^{-1})	Assignment	Reference
Water + Quartz	800	Quartz	(1)
	1064	Quartz	(1)
	1200	Quartz	(1)
	1641	H_2O	(2)
Nucleolus	725	DNA/RNA	(3)
	813	RNA	(4)
	1100	Proteins	(5)
	1322	Nucleic acids	(6)
Lipid droplets	702	Cholesterol esters	(7)
	1064	Lipids	(8)
	1439	Lipids	(9)
	1656	Unsaturated lipids	(5)
Nucleus	748	DNA	(10)
	934	Collagen	(9)
	1340	Nucleic acids	(11)
	1459	Deoxyribose	(12)
Protein enriched regions	850	Amino acids	(13)
	1004	Phenylalanine	(14)
	1208	Protein	(15)
	1240	RNA	(4)

Fused images results from incomplete multiset analysis

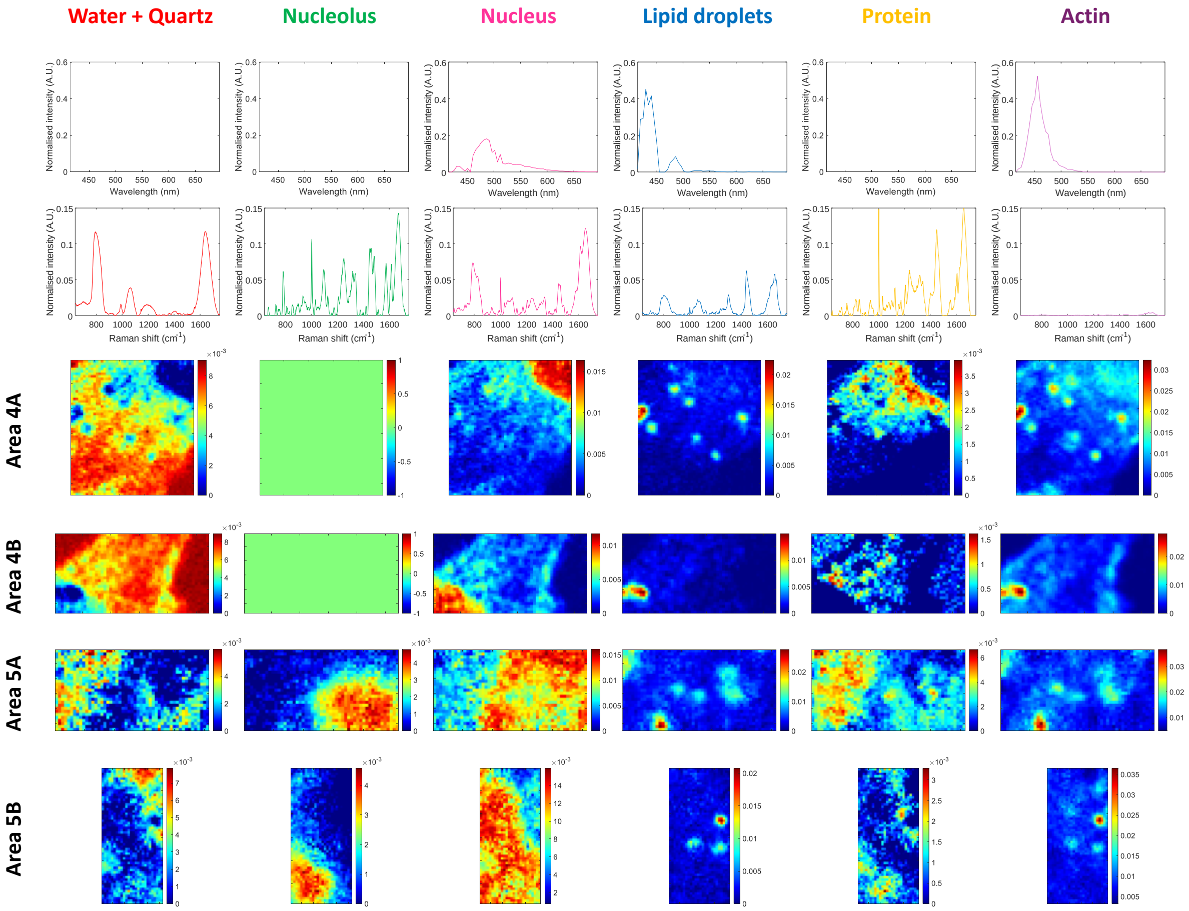

Figure S8. MCR-ALS results of the incomplete multiset analysis. Top: fluorescence and Raman pure spectral profiles of the components. Below: pure distribution maps of the cellular components. Green maps represent absent components in Areas 4A and 4B.

Fluorescence distribution maps from incomplete multiset analysis

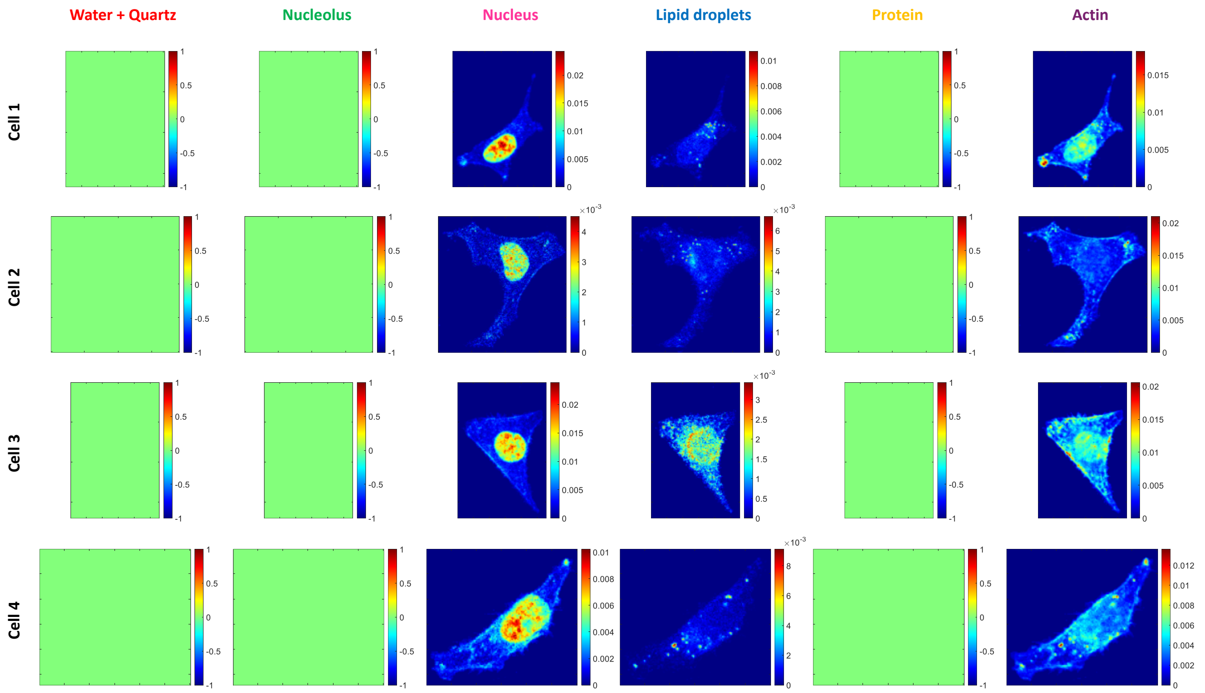

Figure S9. Pure distribution maps of the fluorescence images included in the incomplete multiset analysis. Green maps represent non fluorescent components (unlabelled cellular structures).

References

(1) Mishchik, K. Ultrafast laser-induced modification of optical glasses: a spectroscopy insight into the microscopic mechanisms. **2012**. PhD Thesis. Université Jean Monnet-Saint-Etienne.

(2) Carey, D. M.; Korenowski, G. M. Measurement of the Raman spectrum of liquid water. *J. Chem. Phys.* **1998**, 108(7), 2669-2675.

(3) Chan, J. W.; Taylor, D. S.; Zwerdling, T.; Lane, S. M.; Ihara, K.; Huser, T. Micro-Raman spectroscopy detects individual neoplastic and normal hematopoietic cells. *Biophys. J.* **2006**, 90(2), 648-656.

(4) Notingher, I.; Green, C.; Dyer, C.; Perkins, E.; Hopkins, N.; Lindsay, C.; Hench, L. L. Discrimination between ricin and sulphur mustard toxicity in vitro using Raman spectroscopy. *J. R. Soc. Interface.* **2004**, 1(1), 79-90.

(5) Lakshmi, R. J.; Kartha, V. B.; Murali Krishna, C.; R. Solomon, J. G.; Ullas, G.; Uma Devi, P. Tissue Raman spectroscopy for the study of radiation damage: brain irradiation of mice. *Radiat. Res.* **2002**, 157(2), 175-182.

(6) Huang, Z.; McWilliams, A.; Lam, S.; English, J.; McLean, D. I.; Lui, H.; Zeng, H. Effect of formalin fixation on the near-infrared Raman spectroscopy of normal and cancerous human bronchial tissues. *Int. J. Oncol.* **2003**, 23(3), 649-655.

(7) Krafft, C.; Neudert, L.; Simat, T.; Salzer, R. Near infrared Raman spectra of human brain lipids. *Spectrochim. Acta. Part A.* **2005**, 61(7), 1529-1535.

(8) Stone, N.; Kendall, C.; Smith, J.; Crow, P.; Barr, H. Raman spectroscopy for identification of epithelial cancers. *Faraday Discuss.* **2004**, 126, 141-157.

(9) Frank, C. J.; McCreery, R. L.; Redd, D. C. Raman spectroscopy of normal and diseased human breast tissues. *Anal. Chem.* **1995**, 67(5), 777-783.

(10) Binoy, J.; Abraham, J. P.; Joe, I. H.; Jayakumar, V. S.; Pettit, G. R.; Nielsen, O. F. NIR-FT Raman and FT-IR spectral studies and ab initio calculations of the anti-cancer drug combretastatin-A4. *J. Raman Spectrosc.* **2004**, 35(11), 939-946.

(11) Fung, M. F. K.; Senterman, M. K.; Mikhael, N. Z.; Lacelle, S.; Wong, P. T. Pressure-tuning fourier transform infrared spectroscopic study of carcinogenesis in human endometrium. *Biospectroscopy*. **1996**, 2(3), 155-165.

(12) Ruiz-Chica, A. J.; Medina, M. A.; Sánchez-Jiménez, F.; Ramirez, F. J. Characterization by Raman spectroscopy of conformational changes on guanine–cytosine and adenine–thymine oligonucleotides induced by aminooxy analogues of spermidine. *J. Raman Spectrosc.* **2004**, 35(2), 93-100.

(13) Gniadecka, M.; Wulf, H. C.; Nymark Mortensen, N.; Faurskov Nielsen, O.; Christensen, D. H. Diagnosis of basal cell carcinoma by Raman spectroscopy. *J. Raman Spectrosc.* **1997**, 28(2-3), 125-129.

(14) Cheng, W.-T.; Liu, M.-T.; Liu, H.-N.; Lin, S.-Y. Micro-Raman spectroscopy used to identify and grade human skin pilomatrixoma. *Microsc. Res. Tech.* **2005**, 68(2), 75–79.

(15) Huang, Z.; McWilliams, A.; Lui, M.; McLean, D.I.; Lam, S.; Zeng, H. Near-infrared Raman spectroscopy for optical diagnosis of lung cancer. *Int. J. Cancer.* **2003**, 107, 1047-1052.
